# Psychological nursing intervention on anxiety and depression in patients with urinary incontinence after radical prostatectomy: A randomized controlled study protocol

**DOI:** 10.1097/MD.0000000000023127

**Published:** 2020-11-25

**Authors:** Liying Yang, Danjuan Ling, Lanfen Ye, Manping Zeng

**Affiliations:** a Department of Urology, Taizhou Hospital; b Outpatient Injection Room, Linhai Hospital of Traditional Chinese Medicine, Zhejiang Province; c Department of Traditional Chinese Medicine, First People's Hospital of Chenzhou City, Hunan Province, China.

**Keywords:** anxiety, depression, prostate cancer, protocol, radical prostatectomy

## Abstract

**Background:**

Prostate cancer (PC) is one of the most familiar disease of the male reproductive system globally. In treating the clinically localized PC, the radical prostatectomy is regarded as a gold standard, but it is associated with syndromes as urinary incontinence (UI), which can have a significant impact on patients’ quality of life. Nurse takes responsibility in the management of the UI for their convenience compared with doctors to contact with patients and build better trust relationships with survivals. However, most of the studies focus on the physiological level, the psychological nursing intervention research is less. The purpose of the trial is to introduce a psychological intervention program and to study its effects on anxiety and depression after prostatectomy in IU patients.

**Methods:**

This is a single-center randomized controlled trial that was authorized by Ethics Committee of the First People's Hospital of Chenzhou City (2020054). One hundred participants who undergo radical prostatectomy are analyzed. Inclusion criteria are the following: PC is diagnosed based on histological results; Participants in the study voluntarily sign the informed consent table; Severe UI after extubation; Patients with postoperative UI do not receive any drug treatment. Exclusion criteria are the followings: patients with the history of prostate operation; patients with the history of severe renal and liver malignancy; UI caused by reasons other than prostatectomy. The main outcomes are the degree of anxiety and depression 2 months after urinary catheter is removed. The secondary outcomes are the quality of life 2 months after urinary catheter is removed. All data are collected and analyzed by the Social Science software version 21.0 (SPSS, Inc., Chicago, IL) program.

**Results:**

The relevant indexes of severe UI patients are compared in the table.

**Conclusion:**

Psychological nursing intervention may have a positive effect on depression and anxiety in the UI patients after receiving the radical prostatectomy.

## 1 Introduction

Prostate cancer (PC) is one of the most familiar disease of the male reproductive system globally, with 1,276,106 new cases in 2018, resulting in 358,989 deaths.^[1,2]^ The incidence and mortality rates of PC are associated with aging, with an average age of 66 at the time of diagnosis. Though there will be an estimated 2,293,818 new cases by 2040,^[3]^ there will be a small change in the mortality rate (an increase of 1.05%).

In treating the clinically localized PC, the radical prostatectomy is regarded as a gold standard,^[4]^ but it is associated with syndromes as urinary incontinence (UI), which can have a significant impact on patients’ quality of life.^[5,6]^ The incidence of UI after radical prostatectomy ranges from 4 to 31%, which is related to the clinical characteristics, intraoperative injuries, surgical methods, and surgeon experience.^[7]^ The UI in the early postoperative period is more serious. However, continence recovery may exist in the first 3 to 6 months or later. The problem can lead to feelings of anxiety and depression, which often occur in men after surgery because of the uncertainty about how to deal with those undesirable effects.

Published research reports that physical, psychological, and behavioral factors associated with urinary control affect patients’ quality of life.^[8,9]^ Physiological level is the top topic of the current studies, the psychological nursing intervention research is less. Most of the researches on psychological interventions for UI has focused on women with postpartum incontinence and has little applicability to men. The purpose of the trial is to introduce a psychological intervention program and to research its effects on anxiety and depression in patients with severe IU after prostatectomy.

## 2 Method

### 2.1 Study design

This is a single-center randomized controlled trial that will be implemented from October 2020 to October 2021. The study is conducted in accordance with the psychiatric checklist for randomized studies authorized by the First People's Hospital of Chenzhou City Ethics Committee (2020054) and registered in Research Registry (researchregistry6082).

### 2.2 Subjects

One hundred participants who undergo radical prostatectomy are analyzed. Via utilizing the number table, all the patients involved in the experiment are assigned a random number in a random envelope, and the allocation results are hidden from them. The patients are randomly divided into control group (routine nursing, 50 cases) and study group (psychological education, 50 cases). Inclusion criteria are the following:

(1) PC is diagnosed based on histological results;
(2) Participants in the study voluntarily sign the informed consent table;
(3) Severe UI after extubation;
(4) Patients with postoperative UI do not receive any drug treatment.

Exclusion criteria are the followings:

(1) patients with the history of prostate operation;
(2) patients with the history of severe renal and liver malignancy;
(3) UI caused by reasons other than prostatectomy.

### 2.3 Interventions

Control group receives conventional nursing intervention, including health education to the patients before surgery, correct guidance before and after the urinary catheter is removed, and the health education for the patients after discharge, and conducting the telephone follow-up (containing psychological counseling, the care of daily life, training methods of pelvic floor muscle and bladder).

In addition to the basic routine nursing care, as provided in the routine nursing group, the patients in the psychoeducation group are intervened by a team and 2 nurses with an intermediate professional qualification. The responsibility of doctor is to perform the surgery and the rounds after operation, and to manage the medical conditions caused by surgery. The psychological education group receives the same routine care and health guidance as the routine care group. The duty of the head nurse is to manage the postoperative recovery of patients, actively monitor and care, listen to the complaints of patients in time, and communicate with the patients along with their families regularly, and then actively deal with the related problems of patients. Encourage the patients’ family members to involve in the monitoring and then assistance the relevant training, construct trust relationship with the patients as well as their families, thereby ensuring the smooth implementation of the psychological nursing intervention. After the catheter is removed, the patient is guided through a relaxation exercise that has been learned in advance of the surgery. Patients are followed up by telephone once a week after removal of the catheter. All nursing team members participate in group psychological nursing intervention.

### 2.4 Outcome measures

The main outcomes are the degree of anxiety and depression 2 months after urinary catheter is removed. The secondary outcomes are the quality of life 2 months after urinary catheter is removed. The evaluation of depression degree is carried out with the “Zung self-rating Depression Scale,” while the anxiety degree is evaluated through the “Zung Self-Rating Anxiety Scale.”^[10]^ The assessment of life quality is conducted through applying the specific life quality scale for the UI.^[11]^

### 2.5 Statistical analysis

All data are collected and analyzed by Social Sciences software version 21.0 program. The categorical variables are expressed as percentage of patients and analyzed with the Pearson Chi-square test or the Fisher exact test. The continuous variables could be described as the mean (standard deviation), which are analyzed via the independent *t*-tests or the paired *t*-tests. For the significance level, the value of *P* needed to be less than .05.

## 3 Results

Comparison of indicators related to patients with severe UI is shown in Table 1.

**Table 1:** Comparison of indicators related to patients with severe urinary incontinence.

	Study group (n = 50)	Control group (n = 50)	*P*-value
SAS score			
SDS score			
Restrictive behavior			
Psychosocial influence			
Social barrier			
Quality of life scale			

## 4 Discussion

Radical prostatectomy is the common treatment for PC^[12]^ and the most common postoperative complications are the injury of urethral sphincter and UI caused by parastatal neurovascular bundle.^[13,14]^ UI after operation will not threaten the patients’ life, but will significantly affect the life quality of patients. Patients with severe UI are generally suffered anxiety and depression in the social, psychological as well as physical aspects.^[15,16]^ In fact, UI has already affected the original physiological balance of patients, causing water restriction, lacking sleep, sexual dysfunction, skin eczema and ulcers, reducing the comfort with life, further aggravating the patient's anxiety and depression.^[17,18]^ In addition, UI brings serious economic pressure and psychological distress to the patients and their families, which leads to a sense of shame and loss. In managing the UI, the nurses reveal a significant effect due to they are more likely to establish the better trust relationships with patients. Hence, the purpose of this project is to implement a program of psychological intervention to investigate its effects on depression and anxiety in the severe UI patients after receiving prostatectomy.

## 5 Conclusion

Psychological nursing intervention may have a positive effect on depression and anxiety in the UI patients after receiving the radical prostatectomy.

## Author contributions

**Conceptualization:** Lanfen Ye.

**Data curation:** Lanfen Ye.

**Funding acquisition:** Manping Zeng.

**Investigation:** Danjuan Ling.

**Methodology:** Danjuan Ling.

**Resources:** Lanfen Ye.

**Software:** Lanfen Ye.

**Writing – original draft:** Liying Yang.
